# Increasing Macrolide and Fluoroquinolone Resistance in *Mycoplasma genitalium*

**DOI:** 10.3201/eid2305.161745

**Published:** 2017-05

**Authors:** Gerald L. Murray, Catriona S. Bradshaw, Melanie Bissessor, Jennifer Danielewski, Suzanne M. Garland, Jørgen S. Jensen, Christopher K. Fairley, Sepehr N. Tabrizi

**Affiliations:** Monash University, Clayton, Victoria, Australia (G.L. Murray);; Murdoch Childrens Research Institute, Parkville, Victoria, Australia (G.L. Murray, J. Danielewski, S.M. Garland, S.N. Tabrizi);; Royal Women’s Hospital, Parkville (G.L. Murray, S.M. Garland, S.N. Tabrizi);; Alfred Health, Carlton, Victoria, Australia (C.S. Bradshaw, M. Bissessor**,** C.K. Fairley);; University of Melbourne, Parkville (C.S. Bradshaw, S.M. Garland, S.N. Tabrizi);; Monash University, Melbourne, Victoria, Australia (C.S. Bradshaw, C.K. Fairley);; Statens Serum Institut, Copenhagen, Denmark (J.S. Jensen)

**Keywords:** *Mycoplasma genitalium*, moxifloxacin, ParC, GyrA, antibiotic resistance, antimicrobial resistance, bacteria, Australia

## Abstract

Escalating resistance to azithromycin and moxifloxacin is being reported for *Mycoplasma genitalium* in the Asia-Pacific region. Analyzing 140 infections, we found pretreatment fluoroquinolone-resistance mutations in *parC* (13.6%) and *gyrA* (5%). ParC S83 changes were associated with moxifloxacin failure. Combined macrolide/fluoroquinolone-resistance mutations were in 8.6% of specimens, for which recommended therapies would be ineffective.

*Mycoplasma genitalium* infection is a major cause of urethritis in men and is associated with cervicitis, pelvic inflammatory disease, preterm birth, and spontaneous abortion in women (*1*). In the United States, Australia, and Europe, the recommended first-line treatment for *M. genitalium* infection is the macrolide azithromycin. However, a recent meta-analysis documented a rapid decline in its efficacy, from 85% before 2009 to 67% after 2009; the highest levels of resistance were in the Asia-Pacific region (*2*). The second-line therapy recommended by the US Centers for Disease Control and Prevention (https://www.cdc.gov/std/tg2015/default.htm) is the fluoroquinolone moxifloxacin. Quinolones target the DNA gyrase (comprising GyrA and GyrB) and topoisomerase IV (ParC and ParE). Quinolone binding involves serine at position 83 (*Escherichia coli* GyrA numbering) and the acidic amino acid 4 positions away (D87 or E87) (Technical Appendix) (*3*). Mutations affecting these residues or surrounding sequence (the quinolone resistance-determining region, QRDR) may confer resistance (*4*).

Moxifloxacin treatment failure is being increasingly reported, particularly in the Asia-Pacific region (*5**,**6*), along with increasing detection rates of resistance mutations (*7*). Although several studies have reported the prevalence of QRDR mutations in *M. genitalium*, most mutations have not been linked with treatment outcomes. Our aims with this study were to report the prevalence of mutations in the *parC* and *gyrA* genes in patients with *M. genitalium* infection, to correlate specific mutations with moxifloxacin outcomes, and to determine the prevalence of dual (macrolide/fluoroquinolone) resistance.

## The Study

From July 1, 2012, through June 30, 2013, samples were collected from consecutive *M. genitalium–*infected participants at the Melbourne Sexual Health Centre in Australia (*5*). Detection of *M. genitalium*, load quantitation, and sequence analyses were performed as described previously (*5*,*8*). Overall, 155 patients (112 men, 43 women) with PCR-confirmed *M. genitalium* infection were recruited, representing 90% of patients with infections diagnosed at the Centre over the study period. We obtained adequate samples from 140 of the 155 patients to generate baseline *parC* and *gyrA* gene sequences; these 140 formed the study group.

Patients were initially given a single dose of 1 g azithromycin. The 54 for whom this treatment failed (positive by PCR test-of-cure at day 28 or persistent symptoms before day 28, with no identified reinfection risk) were given moxifloxacin (400 mg/d for 10 d). The 6 for whom moxifloxacin treatment failed were given pristinamycin (1 g 4×/d for 10 d). This study was approved by The Alfred Hospital Ethics Committee (no. 150/12), and informed consent was obtained from patients.

In pretreatment specimens, various single-nucleotide polymorphisms (SNPs) were observed in the *parC* and *gyrA* QRDR (Table 1; Technical Appendix). Of the 19 (13.6%) of 140 samples with ParC substitutions, 16 had S83 mutations (14 S83I, 2 S83R) and 3 had D87N substitutions.

**Table 1:** Pretreatment *gyrA* and *parC* SNPs detected according to moxifloxacin treatment from 140 samples collected from patients with *Mycoplasma genitalium* infection, Melbourne, Australia, July 2012 through June 2013*

Gene, SNP†	Amino acid change	Moxifloxacin failure, no. (%), n = 6‡	Moxifloxacin success, no. (%), n = 48	Not treated with moxifloxacin, no. (%), n = 86	Total prevalence, no. (%), n = 140
*gyrA*					
A229G§	K77E	–	–	1 (1.2)	1 (0.71)
G240A§	Silent (R80)	–	–	1 (1.2)	1 (0.71)
G285A	M95I	2 (33)¶	2 (4.2)¶	–	4 (2.9)
G295A§	D99N	1 (17)¶	–	–	1 (0.71)
G295T§	D99Y	1 (17)¶	–	–	1 (0.71)
A296G§	D99G	1 (17)	–	–	1 (0.71)
*parC*					
C184T§	P62S	–	–	13 (15)	13 (9.3)
C234T	Silent (H78)	–	6 (12.5)	10 (12)	17 (12)
A247C	S83R	2 (33)	–	–	2 (1.4)
G248T	S83I	4 (67)	3 (6.3)	7 (8.1)	14 (10)
G259A	D87N	–	1 (2.1)	2 (2.3)	3 (2.1)
T269A§	I90N	–	–	1 (1.2)	1 (0.71)
C324T§	Silent (N108)	–	–	1 (1.2)	1 (0.71)
No change	–	–	38 (79)	60 (70)	98 (68.5)

*SNP, single-nucleotide polymorphism; –, mutation absent. †The *M. genitalium* G37 genome sequence (NC_000908) was the reference sequence. ‡All corresponding postmoxifloxacin treatment-failure samples contained the same SNP profiles. Multiple SNPs were detected in some samples. §Sequence variations not previously described in *M. genitalium.* ¶A mutation was combined with an S83 change.

We found a significant association between detection of ParC S83 mutations and treatment failure. *M. genitalium* from all 6 patients for whom moxifloxacin failed but from only 3 of the 48 patients for whom moxifloxacin was effective had the ParC S83 mutation (p<0.0001 by Fisher exact test) (Table 2, patients 1–9). The 3 infections successfully treated despite a change at ParC S83 are of interest. For these patients, low bacterial load may have contributed to therapeutic success (*9*), led to spontaneous clearance, or resulted in false-negative follow-up PCR (Table 2). However, in contrast, treatment failed for 1 patient with a low anal load of *M. genitalium* and S83 change. Similar to the findings for this study, in the parent cohort of 155 patients, organism load influenced apparent azithromycin cure; 7% of infections carrying markers of azithromycin resistance were cured by azithromycin, and organism load was significantly lower than that among those with resistant infections for whom azithromycin treatment failed (*5*).

**Table 2:** Samples containing changes in key amino acids of ParC and GyrA from 140 patients *with Mycoplasma genitalium* infection, Melbourne, Australia, July 2012 through June 2013*

Patient no./sex	Sample	Log_10_ load, GEQ†	Treatment (moxifloxacin) outcome	SNPs in *parC*				SNPs in *gyrA*			
				A247C S83R	G248T S83I	G259A D87N		G285A M95I	G295A D99N	G295T D99Y	A296G D99G
1/F	Cervical swab	4.73	Failure	–	+	–		+	–	–	–
2/M	Urine	2.94	Failure	–	+	–		+	–	–	–
3/F	Anal swab	2.15	Failure	–	+	–		–	+	–	–
4/M	Urine	3.80	Failure	–	+	–		–	–	–	+
5/M	Urethral swab	5.10	Failure	+	–	–		–	–	+	–
6/M	Urine	4.66	Failure	+	–	–		–	–	–	–
7/F	Cervical swab	1.82	Success	–	+	–		+	–	–	–
8/M	Urine	2.47	Success	–	+	–		+	–	–	–
9/M	Urine	1.82	Success	–	+	–		–	–	–	–
10/M	Urine	4.07	Success	–	–	+		–	–	–	–
11/F	Urine	2.47	NT	–	+	–		–	–	–	–
12/M	Urine	3.46	NT	–	+	–		–	–	–	–
13/M	Urine	4.51	NT	–	+	–		–	–	–	–
14/M	Urine	3.51	NT	–	+	–		–	–	–	–
15/M	Urine	2.25	NT	–	+	–		–	–	–	–
16/M	Urine	1.11	NT	–	+	–		–	–	–	–
17/F	Urine	1.94	NT	–	+	–		–	–	–	–
18/M	Urine	2.50	NT	–	–	+		–	–	–	–
19/F	Cervical/swab	3.08	NT	–	–	+		–	–	–	–

*Dataset includes all failed treatments for moxifloxacin. GEQ, genome equivalent; NT, not treated with moxifloxacin because azithromycin treatment was successful; SNP, single-nucleotide polymorphism; +, sequencing of sample successful and SNP present; –, SNP absent. †The log_10_ loads of *M. genitalium* were calculated per swab or 1 mL of urine. For all 155 patients in the parent cohort, loads varied from 0.84 to 6.17 (median 3.36).

The prevalence of S83 changes in this study is higher than that detected in a study at Sydney Sexual Health Centre (Sydney, New South Wales, Australia) (8.4%, n = 143) (*10*). Studies in Japan reported prevalence ranging from 3.6% (n = 28) to 29.4% (n = 51) and 36.8% (n = 19) (*7**,**8**,**11*), although 1 study involved a cohort at higher risk (female sex workers). A low prevalence of S83 mutation has been observed in Europe (1.5% in France, 5% in England and Germany) (*12**–**14*). This mutation has been associated with moxifloxacin failure in 3/3 cases in the Sydney-based study (*6*).

SNPs that changed the ParC acidic residue (D87) were rare (2.1%) and because of low numbers could not be associated with treatment outcomes. Other studies found higher frequency of this change (3.5%–7.1%) (*7*,*8**,**10**,**15*); authors of 1 study reported an association with levofloxacin failure (*15*).

*M. genitalium* GyrA lacks the S83 residue common to GyrA of other bacteria, having instead a methionine at the equivalent position (M95). This enzyme is therefore probably partially resistant to quinolones. GyrA changes (at M95 or D99) occurred at a frequency of 5.0% (7/140) but could not be correlated with treatment outcome because they occurred concurrently with S83 changes in ParC. Previously, a GyrA M95I change was associated with *M. genitalium* treatment failure in 1 patient (*6*).

Patients who received moxifloxacin were followed up with a PCR test-of-cure at 14 and 28 days. For the 6 for whom treatment failed, the mutation profiles in follow-up specimens were unchanged from the initial premoxifloxacin sequence, suggesting lack of resistance selection in vivo after moxifloxacin.

A total of 60 (42.9%) of the 140 pretreatment samples had macrolide-resistance mutations (*5*). Both macrolide and *parC* fluoroquinolone mutations at S83 or D87 were present in 12 (8.6%) of the 140 samples. Prevalence of fluoroquinolone resistance markers was higher in samples with (20%, 12/60) than without (8.8%, 7/80) macrolide-resistance mutations, although this difference did not reach statistical significance (p = 0.08). This finding suggests that successive treatment failures with first-line, then second-line, antimicrobial drugs are generating strains resistant to 2 classes of drugs. Previous studies found lower levels of combined macrolide and fluoroquinolone mutations in men attending a urology clinic (3/51, 5.9%) (*7*) and higher levels in a high-risk population (female sex workers; 4/16, 25%) (*11*).

This study has limitations. The resistance profiles for the infecting strains of *M. genitalium* were not tested in in vitro culture. There may be other unknown changes in the genome that confer resistance to the drugs of interest. In addition, the resistance levels reported are probably underestimates because samples were collected in 2012–2013 and levels have probably risen since then (*7*).

## Conclusions

We found high frequency of ParC S83 changes associated with fluoroquinolone resistance in a sexually transmitted infection clinic in urban Australia; these changes were associated with moxifloxacin failure. The high level of dual markers for macrolide/fluoroquinolone resistance suggests successive treatment failure after sequential monotherapy leading to the serious outcome that ≈10% of *M. genitalium* infections are not treatable with recommended or readily available antimicrobial drugs. In the absence of alternatives, treatment with pristinamycin cured all 6 patients with dual-class resistance infections (G.L. Murray et al., unpub data).

This study highlights the urgent need for antimicrobial drug resistance surveillance and the value of diagnostic assays that report the presence of resistance markers to optimize treatment. Our results suggest that it is time to reconsider the indications for azithromycin and invest in trials of different available as well as novel classes of antimicrobial drugs for *M. genitalium* treatment. They also raise serious concerns about sequential use of monotherapy and the need to evaluate combination therapies as we enter a new era of untreatable sexually transmitted infections.

Technical Appendix Comparison of amino acid sequence changes in *Mycoplasma genitalium* ParC and GyrA.
