# Supplementary material for: Increasing Macrolide and Fluoroquinolone Resistance in Mycoplasma genitalium
Source: Emerg Infect Dis. 2017 May;23(5):809–12. doi: 10.3201/eid2305.161745 (PMC5403035; doi:10.3201/eid2305.161745)
Supplement: Technical Appendix — Comparison of amino acid sequence changes in Mycoplasma genitalium ParC and GyrA. [file 16-1745-Techapp-s1.pdf]

Article DOI: http://dx.doi.org/10.3201/eid2305.161745

Macrolide and Fluoroquinolone Resistance in *Mycoplasma genitalium*

Technical Appendix

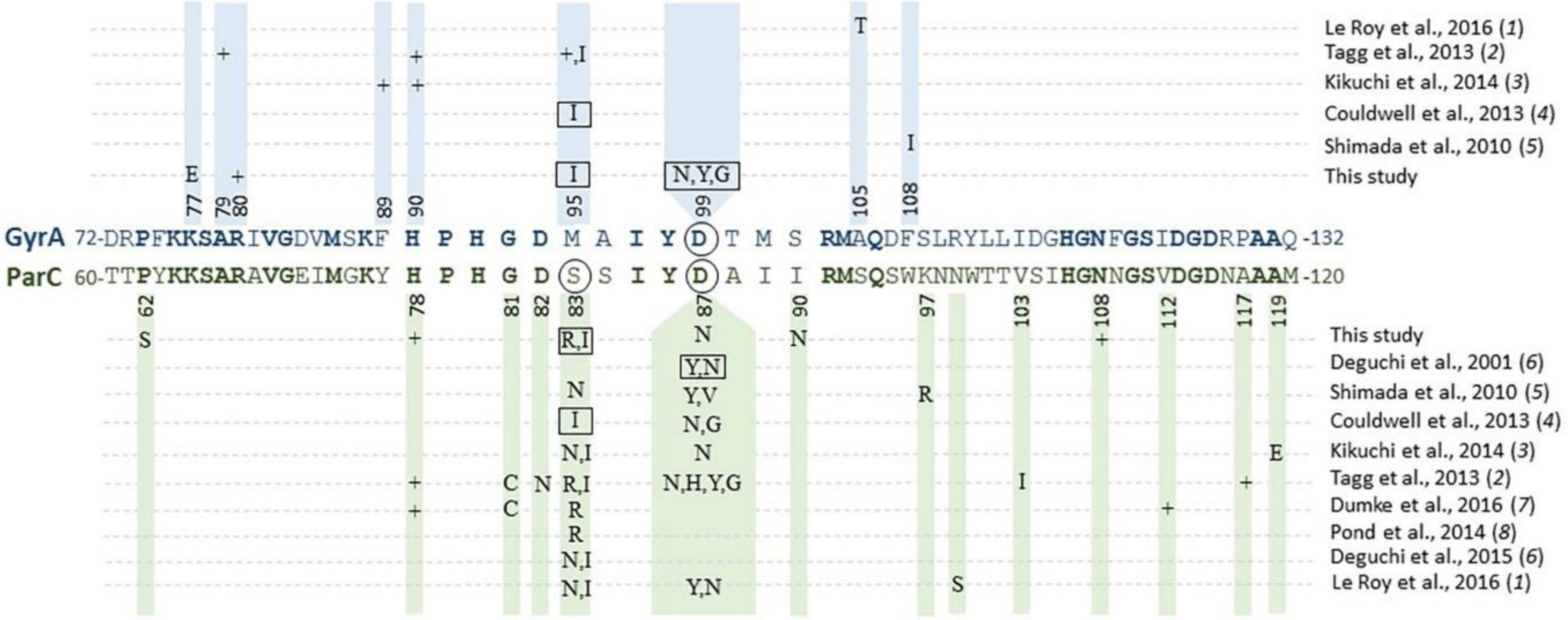

Technical Appendix Figure. Comparison of amino acid sequence changes in *Mycoplasma genitalium* ParC and GyrA. The amino acid sequences from strain G37 were aligned, and the relevant portion of the sequence is presented, with identical residues indicated in boldface. Numbers refer to the amino acid sequence number from the *M. genitalium* sequence. Changes in sequence at specific residues are indicated by

letters enclosed by shading, either above the alignment (GyrA) or below (ParC). The relevant study is indicated on the right. A boxed letter indicates that the change has been linked with fluoroquinolone treatment failure. Circled amino acids in the primary sequences indicate residues that may be directly involved in fluoroquinolone binding. + indicates silent mutation.

References

1. Le Roy C, Hénin N, Pereyre S, Bébéar C. Fluoroquinolone-resistant *Mycoplasma genitalium*, southwestern France. Emerg Infect Dis. 2016;22:1677–9. PubMed http://dx.doi.org/10.3201/eid2209.160446

2. Tagg KA, Jeoffreys NJ, Couldwell DL, Donald JA, Gilbert GL. Fluoroquinolone and macrolide resistance–associated mutations in *Mycoplasma genitalium*. J Clin Microbiol. 2013;51:2245–9. PubMed http://dx.doi.org/10.1128/JCM.00495-13

3. Kikuchi M, Ito S, Yasuda M, Tsuchiya T, Hatazaki K, Takanashi M, et al. Remarkable increase in fluoroquinolone-resistant *Mycoplasma genitalium* in Japan. J Antimicrob Chemother. 2014;69:2376–82. PubMed http://dx.doi.org/10.1093/jac/dku164

4. Couldwell DL, Tagg KA, Jeoffreys NJ, Gilbert GL. Failure of moxifloxacin treatment in *Mycoplasma genitalium* infections due to macrolide and fluoroquinolone resistance. Int J STD AIDS. 2013;24:822–8. PubMed http://dx.doi.org/10.1177/0956462413502008

5. Shimada Y, Deguchi T, Nakane K, Masue T, Yasuda M, Yokoi S, et al. Emergence of clinical strains of *Mycoplasma genitalium* harbouring alterations in ParC associated with fluoroquinolone resistance. Int J Antimicrob Agents. 2010;36:255–8. PubMed http://dx.doi.org/10.1016/j.ijantimicag.2010.05.011

6. Deguchi T, Maeda S, Tamaki M, Yoshida T, Ishiko H, Ito M, et al. Analysis of the *gyrA* and *parC* genes of *Mycoplasma genitalium* detected in first-pass urine of men with non-gonococcal urethritis before and after fluoroquinolone treatment. J Antimicrob Chemother. 2001;48:742–4. PubMed http://dx.doi.org/10.1093/jac/48.5.742

7. Dumke R, Thurmer A, Jacobs E. Emergence of *Mycoplasma genitalium* strains showing mutations associated with macrolide and fluoroquinolone resistance in the region Dresden, Germany. Diagn Microbiol Infect Dis. 2016;86:221–3. PubMed http://dx.doi.org/10.1016/j.diagmicrobio.2016.07.005

8. Pond MJ, Nori AV, Witney AA, Lopeman RC, Butcher PD, Sadiq ST. High prevalence of antibiotic-resistant *Mycoplasma genitalium* in nongonococcal urethritis: the need for routine testing and the inadequacy of current treatment options. Clin Infect Dis. 2014;58:631–7. http://dx.doi.org/10.1093/cid/cit752
